# Cost-consequence analysis of a criterion-based screening and triage pathway compared with full ADHD assessment for adults referred to NHS secondary care

**DOI:** 10.3389/fpsyt.2026.1884417

**Published:** 2026-08-03

**Authors:** Marios Adamou, Ellen Wright

**Affiliations:** 1 School of Human and Health Sciences, University of Huddersfield, Huddersfield, United Kingdom; 2 South West Yorkshire Partnership Teaching National Health Service (NHS) Foundation Trust, Wakefield, United Kingdom

**Keywords:** ADHD, adult ADHD, band 7 clinician, conditional cost-minimisation, cost-consequence analysis, economic assessment, NHS, screening

## Abstract

**Background:**

National evidence indicates that attention-deficit hyperactivity disorder (ADHD) referrals, recorded diagnoses and waiting lists have increased substantially. The Comprehensive ADHD Screening Questionnaire (CASQ), a criterion-based tool delivered by trained Band 7 clinicians, achieved 100% sensitivity, with a 95% confidence interval of 61.0–100.0%, while screening out 39.6% of referrals in a prospective NHS validation study of 48 participants. This analysis compares the costs and diagnostic consequences of the CASQ-first pathway with full ADHD assessment from the NHS perspective.

**Methods:**

A prospective observational study-based cost-consequence analysis was conducted from the NHS perspective over a one-year time horizon, following the Consolidated Health Economic Evaluation Reporting Standards (CHEERS) 2022. A non-inferiority margin of −10 percentage points, corresponding to sensitivity of at least 90%, was specified to assess diagnostic safety. The CASQ assessment-and-triage cost was £120.27 per referral, derived bottom-up from Personal Social Services Research Unit (PSSRU) 2024 unit costs. The standard or low-complexity full ADHD assessment cost was £850, taken from the final NHS England 2026/27 Adult ADHD Assessment, Face-to-Face guide price. The weighted-mean full ADHD assessment cost was calculated using South West Yorkshire Partnership Teaching NHS Foundation Trust (SWYPFT) case-mix proportions from a previous explainable clustering analysis, giving £1, 011.28 per referral. Sensitivity analyses examined alternative cost assumptions, proportion screened out, CASQ duration and incremental clinical time for more complex full ADHD assessment cases. An expanded structural probabilistic sensitivity analysis with 10, 000 Monte Carlo simulations separately propagated uncertainty in complexity-cluster proportions, incremental Band 7 clinical hours, CASQ assessment-and-triage cost, standard full ADHD assessment cost, Band 7 hourly cost and diagnostic accuracy parameters.

**Results:**

The CASQ achieved 100% sensitivity and 100% negative predictive value in the validation study. In the SWYPFT case-mix weighted-mean base case, the CASQ-first pathway cost £731.08 per referral compared with £1, 011.28 for universal full ADHD assessment, a saving of £280.20 per referral and £598, 507 annually at 2, 136 referrals. In commissioning terms, this corresponds to an estimated saving of approximately £280, 200 for every 1, 000 referrals managed through the CASQ-first pathway under the SWYPFT case-mix weighted base case. In the expanded structural probabilistic sensitivity analysis, the CASQ-first pathway was cost-saving in 100.0% of 10, 000 simulations, with a mean saving of £285.18 per referral and £609, 143 annually at 2, 136 referrals. CASQ sensitivity met the specified 90% sensitivity threshold in 75.0% of simulations. The mean projected false-negative rate was 9.4 per 1, 000 referrals.

**Conclusion:**

The CASQ-first pathway may reduce assessment costs and redirect a substantial proportion of referrals away from a direct full ADHD assessment, but the present validation study is not adequately powered to support a definitive economic conclusion. Because the economic result rests on a sensitivity estimate derived from only six confirmed ADHD cases, the projected per-referral and national savings are illustrative planning estimates and not established savings. The economic estimates presented here are preliminary and conditional and should be regarded as a commissioning planning aid that becomes interpretable only when independent multi-site validation has established a reliable diagnostic safety margin. Decision-makers should weigh the estimated per-referral cost reduction against diagnostic accuracy uncertainty and potential harm to missed cases. Independent multi-site replication with formal non-inferiority testing is required before unconditional cost-minimisation conclusions can be drawn.

## 1 Introduction

### 1.1 The demand challenge for adult ADHD services

Adult ADHD assessment has become one of the most frequently requested functions in NHS mental health services, with current demand pressures increasingly described as a service crisis (1). Historically conceptualised as a childhood condition, ADHD often persists into adulthood (2), and population estimates suggest that adult ADHD affects a clinically significant minority of the population, with a pooled prevalence of 2.5% (3). The interim report of a recently constituted UK Government Independent Review into mental health conditions, ADHD and Autism found that ADHD service demand has risen sharply, but also emphasised that service demand, recorded diagnosis and underlying population prevalence should not be treated as equivalent (4). This suggests that the current level of demand may skew recorded administrative prevalence. The same report noted that population prevalence appears broadly stable compared with the much faster rise in referrals, diagnoses and waiting lists. This distinction is important for service planning, because services should expect a substantial proportion of referrals not to result in a confirmed ADHD diagnosis.

NHS England’s Management Information for ADHD, drawn from the Mental Health Services Dataset (MHSDS), recorded an average of approximately 21, 150 new referrals per month for suspected ADHD across calendar year 2025, compared with approximately 16, 100 in December 2024 (5). Total open referrals reached 562, 480 by 31 December 2025, having grown from 365, 590 a year earlier, a 54% increase in twelve months and more than 18 times the 31, 000 recorded in April 2019. Adults aged 25 years or over accounted for 292, 425, or 52%, of the open referrals at end-2025, and the same publication estimates that approximately 2.50 million people in England have ADHD as of February 2026, of whom 1.61 million are aged 25 years or over.

The clinical consequences of delayed ADHD diagnosis are considerable. Timely recognition and treatment are clinically important because adults with ADHD experience functional impairment across occupational, educational and interpersonal domains, alongside increased risks of accidents, substance misuse and comorbid psychiatric conditions (6–12). Economic analyses indicate that untreated adult ADHD is associated with substantial costs at both individual and societal levels, extending beyond specialist mental health services into employment, productivity, healthcare, accidents, substance misuse and criminal justice domains (13–19). Taken together, this literature supports the view that delayed or missed ADHD diagnosis may displace costs across wider public-sector and societal systems, although total healthcare resource utilisation costs alone may increase after ADHD diagnosis because identification can appropriately activate treatment and monitoring pathways (20).

### 1.2 The comprehensive ADHD screening questionnaire pathway

South West Yorkshire Partnership Teaching NHS Foundation Trust (SWYPFT) implemented a criterion-based Single Point of Access model in January 2025, based on the Comprehensive ADHD Screening Questionnaire. Its full validation is described in the companion validation paper (21). In summary, CASQ is a clinician-administered structured triage tool based on Diagnostic and Statistical Manual of Mental Disorders, Fifth Edition (DSM-5) criteria. It is designed for delivery by trained clinicians operating within local clinical governance arrangements in approximately 60 minutes, comprising 45 minutes of face-to-face assessment and 15 minutes of clinical administration. The tool assesses each of the five DSM-5 diagnostic criteria and generates a binary triage recommendation: Appropriate for full ADHD assessment, or Not Appropriate with signposting to alternative services. A review of the published literature identified no formal validation of a criterion-based clinical triage pathway for adult ADHD in NHS secondary care before the development of CASQ. The purpose of CASQ is not to replace full diagnostic assessment, but to identify which referrals are likely to require full specialist assessment and which may be more appropriately directed to other support. CASQ is therefore a pathway-allocation intervention, not a substitute for full ADHD assessment.

In the companion prospective observational validation study, 48 consecutive adults referred for specialist ADHD assessment at SWYPFT between January 2024 and February 2025 were assessed using CASQ and were also offered a NICE-compliant diagnostic assessment using the Diagnostic Interview for ADHD in Adults **(DIVA-2)** and a full psychiatric history, regardless of the CASQ triage recommendation (21, 22). Six of 48 participants received a confirmed ADHD diagnosis. CASQ correctly identified all six ADHD cases, giving observed sensitivity of 100.0%, with a 95% confidence interval of 61.0–100.0%. No false negatives were observed in the validation sample. CASQ classified 19 referrals, or 39.6%, as Not Appropriate for full ADHD assessment. The negative predictive value was 100.0%, with a 95% confidence interval of 83.2–100.0%, meaning that no person with ADHD was incorrectly excluded in the validation sample.

Although these findings are encouraging, they are from a single-site study with a small sample and limited precision around the sensitivity estimate. CASQ identified all ADHD cases in the validation sample, but only six ADHD-positive cases were present, and the confidence interval around sensitivity remains wide. The validation study therefore cannot yet establish, with sufficient precision, that a CASQ-first pathway has diagnostic safety comparable with a pathway in which all people referred proceed directly to full diagnostic assessment. The CASQ should not be interpreted as equivalent to full diagnostic assessment. Its function is to support pathway allocation by identifying referrals in which ADHD is not sufficiently supported to justify full ADHD assessment, while directing uncertain or potentially ADHD-positive cases to full diagnostic assessment. A definitive cost-minimisation conclusion would therefore be premature. The primary economic analysis is therefore framed as a cost-consequence analysis, with cost-minimisation presented only as a conditional interpretation if future adequately powered studies confirm comparable diagnostic safety.

### 1.3 Objectives

This analysis compares the total NHS cost per referral and the diagnostic consequences of the CASQ-first pathway with those of a full diagnostic assessment for adults referred for specialist ADHD services over a one-year time horizon. The primary analysis is a cost-consequence analysis in which costs and outcomes are reported separately. A secondary conditional cost-minimisation scenario is presented for contexts in which diagnostic non-inferiority is assumed.

## 2 Methods

### 2.1 Health economic analysis plan

No health economic analysis plan was registered before this analysis took place. This cost-consequence analysis was conducted using data from the prospective observational validation study (21) and published NHS unit cost sources (23, 24). Reporting follows the Consolidated Health Economic Evaluation Reporting Standards (CHEERS) 2022 (25), the National Institute for Health and Care Excellence (NICE) reference case for economic assessments in England (26), and International Society for Pharmacoeconomics and Outcomes Research (ISPOR) good-practice recommendations (27). A CHEERS 2022 compliance table is provided in **Appendix B**.

### 2.2 Study population

The population included in the analysis was adults aged 18 years or over referred for their first specialist ADHD assessment, with no previous ADHD diagnosis, as enrolled in the CASQ validation study (21). Participants were referred from primary care to NHS specialist secondary care services. In that validation sample, six of 48 participants received an ADHD diagnosis, giving an observed rate of positive diagnosis of 12.5%. This figure refers to the rate within the CASQ study sample and is not a population prevalence estimate. It reflects the proportion of participants who received an ADHD diagnosis after all cases were offered full ADHD assessment for validation purposes, including those who would not normally have proceeded to full assessment.

The 48 participants represent the validation sample assessed during the study window and do not constitute an annual referral count. They are distinct both from the local annual referral volume of 2, 136 used later to scale per-referral costs into a local budget impact (Section 2.9) and from the national monthly referral volumes reported in Section 1.1. The 12.5% within-sample diagnostic rate is lower than the diagnostic rate that would be expected in routine practice, because the validation design required full ADHD assessment of every referral, including those who would ordinarily have been screened out before assessment. It therefore reflects the validation design of the screening and triage pathway rather than the service-level diagnostic rate.

### 2.3 Setting and location

The analysis reflects NHS resource use in England during 2023/24–2026/27. The clinical setting is NHS specialist secondary care adult neurodevelopmental or mental health services receiving referrals from primary care. Costs are intended to be applicable to NHS secondary care services delivering specialist ADHD assessment under similar pathway and tariff assumptions.

### 2.4 Comparators

Comparator 1 is the CASQ-first pathway. All referrals receive a 60-minute CASQ assessment delivered by a trained Band 7 clinician, consisting of 45 minutes of face-to-face assessment and 15 minutes of clinical administration. Those classified as Appropriate, 60.4% in the validation cohort, proceed to a full ADHD assessment pathway. Those classified as Not Appropriate, 39.6%, are signposted to alternative services and do not proceed to specialist ADHD assessment. Referrals classified as Not Appropriate are discharged from the ADHD assessment pathway and signposted to appropriate alternative support, as described in the companion validation study (21). The CASQ assessment-and-triage cost is £120.27 per referral, derived bottom-up from PSSRU 2024 unit costs (24). It consists of one Band 7 client contact hour at £84, one administrative outcome letter at £12, and £24.27 of wider implementation and service-delivery costs covering referral administration, appointment booking, clinical supervision, clinical governance, audit and quality assurance, information governance, non-attendance management, managerial overhead, initial training, refresher training and service set-up.

Comparator 2 is full ADHD assessment. All referrals proceed directly to a full ADHD assessment pathway. In accordance with the Adult ADHD Assessment Quality Assurance Standard (AQAS), a compliant full ADHD assessment would consist of a semi-structured clinical interview systematically assessing DSM-5 ADHD symptom criteria, structured assessment of functional impairment across domains, detailed developmental history, screening for psychiatric comorbidities and differential diagnoses, and physical health assessment including baseline cardiovascular assessment (28, 29).

The full ADHD assessment pathway is costed at the NHS England 2026/27 Outpatient Guide Price for Adult ADHD Assessment, Face-to-Face: £850 per pathway (23). This is the final 2026/27 guide price and is used throughout. The £850 price is a pathway-level commissioning guide price, not a pure staff-time cost. The NHS England guidance describes the Adult ADHD Assessment currency as including triage of referral, administration costs, psychoeducation, ADHD diagnostic assessment and outcome report, discharge of the patient, and re-engagement following non-attendance. The guidance does not provide a bottom-up derivation of the £850 price by staff grade, clinical time, administrative time, overheads, estates, capital costs or professional mix, and the present analysis does not present such a decomposition. The £850 price is therefore treated as a commissioning-cost anchor for the standard or low-complexity full ADHD assessment pathway and is not mechanically scaled by assessment duration.

The NHS England 2026/27 Adult ADHD Assessment guide price was used as the standard assessment-cost anchor because it is the only nationally specified pathway-level commissioning price for adult ADHD assessment. The guide price is not stratified by clinical complexity and should not be interpreted as a direct staff-time cost or as a tariff explicitly assigned to low-complexity cases. For modelling purposes, it was therefore treated as the unstratified standard assessment cost. In the stratified scenario, low-complexity referrals were assumed to require no additional assessment resource beyond this standard assessment-cost anchor, whereas medium- and high-complexity referrals were modelled by adding incremental Band 7 clinical time. This approach preserves the national commissioning price as the common comparator while allowing plausible additional resource use to be represented for more complex cases.

### 2.5 Perspective, time horizon and discount rate

The analysis was conducted from the NHS and Personal Social Services perspective in accordance with the NICE reference case (26). All resource categories relevant to NHS service delivery are included. Personal Social Services costs, out-of-pocket costs to patients and indirect productivity costs are excluded from the base case but are discussed as wider system considerations. The time horizon is 12 months. Discounting is not applied because all costs are incurred within the one-year time horizon.

### 2.6 Justification of the cost-consequence framework

A cost-minimisation analysis is appropriate only where two or more interventions have been demonstrated to produce equivalent health outcomes (30–32). In the present context, the relevant outcome is diagnostic safety, operationalised as sensitivity. The CASQ-first pathway is not intended to replace full ADHD assessment, but to identify referrals that are unlikely to require it. For the purpose of this pathway comparison, an AQAS-compliant full ADHD assessment was treated as the reference strategy with no triage-related false negatives, because no referred individual is excluded before full ADHD assessment. The CASQ-first pathway achieved 100.0% observed sensitivity in the validation study. However, the 95% confidence interval was wide because the sample was small and included only six ADHD-positive cases. A true sensitivity materially below the observed estimate would mean that an unacceptable proportion of people with ADHD could be incorrectly screened out before full ADHD assessment. The present evidence therefore supports cost-consequence analysis as the primary economic framework, rather than definitive cost-minimisation analysis.

A non-inferiority margin of −10 percentage points relative to full ADHD assessment was clinically specified for diagnostic safety. This margin represents the largest reduction in sensitivity that would be considered clinically acceptable in exchange for the CASQ-first pathway’s potential operational advantages. Because the full ADHD assessment pathway was treated as the reference strategy with no triage-related false negatives, the −10 percentage-point margin required CASQ to demonstrate sensitivity of at least 90%. Although the observed sensitivity in the validation sample was 100.0%, the confidence interval remained too wide to establish non-inferiority with sufficient precision. Cost-minimisation is therefore presented only as a conditional interpretation, dependent on future adequately powered studies confirming comparable diagnostic safety.

### 2.7 Measurement and valuation of resource use and costs

The included resource categories are CASQ delivery by trained Band 7 clinicians, the administrative outcome letter, the wider implementation and service-delivery overhead required to run CASQ as an NHS service, and the full ADHD assessment pathway. Downstream costs such as pharmacological treatment, psychological intervention, monitoring and general practice contacts are assumed equivalent across comparators, conditional on equivalent diagnostic accuracy, and are therefore excluded from both arms.

CASQ delivery is costed using the PSSRU Unit Costs of Health and Social Care 2024, which reports £84 per client contact hour for a Band 7 NHS mental health professional (24). Each CASQ assessment takes 45 minutes of face-to-face clinical interview and 15 minutes of administrative synthesis, totalling one client contact hour. The CASQ delivery cost is therefore £84 per referral. The administrative outcome letter is costed at £10 of Band 4 administrative time plus £1 per letter for postage, with two letters issued per outcome, giving a total outcome-letter cost of £12 per referral. Wider CASQ implementation and service-delivery costs are added bottom-up using PSSRU 2024 unit costs applied to the staff time and recurrent activity required to deliver CASQ as a service. The total wider implementation and service-delivery cost is £24.27 per referral. The CASQ assessment-and-triage cost is therefore £120.27 per referral.

The full ADHD assessment pathway is costed at the NHS England 2026/27 Outpatient Guide Price for Adult ADHD Assessment, Face-to-Face: £850 per pathway (23). Where additional clinical time is required for more complex cases, that time is added incrementally as Band 7 client contact hours on top of the £850 commissioning anchor. The medium-complexity scenario is costed at £1, 018, based on £850 plus two additional Band 7 client contact hours. The high-complexity scenario is costed at £1, 186, based on £850 plus four additional Band 7 client contact hours. The base-case full ADHD assessment cost is the SWYPFT case-mix weighted mean of these three component costs, giving £1, 011.28 per referral.

### 2.8 Currency, price date and conversion

All costs are reported in Great British Pounds sterling. CASQ delivery costs are derived from PSSRU 2024, in 2023/24 prices. The base-case comparator cost is drawn from NHS England 2026/27 Outpatient Guide Prices, in 2026/27 prices (23). No currency conversions are required. No inflation adjustment has been applied to the CASQ-specific staff costs because the difference in price years is unlikely to materially affect conclusions given the modest magnitude of CASQ-specific costs relative to the comparator commissioning anchor.

### 2.9 Rationale and description of model

This is a prospective observational study-based cost-consequence comparison. No modelling extrapolation is applied beyond the local budget-impact scenario. Resource-use quantities are taken from the CASQ validation study: 60.4% proceed to full ADHD assessment and 39.6% are screened out. Unit costs are applied to these quantities to generate a per-referral cost for each comparator. Annual costs are obtained by multiplying per-referral costs by the local projected annual referral volume of 2, 136. This local volume is used for budget-impact modelling only and does not represent a prevalence estimate. This volume is a local service-planning figure; it is not derived from the 48-participant validation sample and is not comparable with the national monthly referral counts reported in Section 1.1. It is used solely to convert per-referral costs into a local annual budget impact.

The per-referral cost calculation assumes that every referral in the full diagnostic pathway proceeds to full ADHD assessment; every referral in the CASQ-first pathway receives a CASQ assessment; 60.4% of CASQ-assessed referrals proceed to full ADHD assessment; 39.6% do not proceed; and no treatment switching between comparators occurs within the time horizon.

The CASQ-first pathway cost per referral is calculated as:


$$C_{CASQ-first}=C_{CASQ}+p_{proceed}\times C_{full\;assessment}$$

where *C_CASQ_* = £120.27, *p_proceed_* = 0.604, *C_full assessment_* = £1, 011.28 and the incremental cost is Δ*C* = *C_CASQ-first_* − *C_full assessment_*.

### 2.10 Characterising heterogeneity

A further source of heterogeneity concerns clinical complexity, which was modelled drawing on previous work in the same service (33). Adult ADHD referral populations are clinically heterogeneous, at least due to comorbidity (34), and the resource required for full ADHD assessment is unlikely to be the same across all cases. The £850 NHS England guide price is treated in this analysis as the cost of the standard or low-complexity full ADHD assessment pathway. For medium- and high-complexity pathways, additional Band 7 client contact hours are added incrementally to the £850 commissioning anchor.

In a previous analysis, explainable clustering was applied to routinely collected pre-assessment data from 786 consecutive adult ADHD referrals (33). This work identified three complexity clusters. A low-complexity cluster consisted of approximately 30.2% of referrals and was characterised by relatively focused ADHD-typical symptoms with limited psychiatric comorbidity, modest functional impairment and minimal differential diagnostic complexity. A medium-complexity cluster comprised approximately 43.6% of referrals and was characterised by moderate symptom burden alongside higher rates of affective symptoms and broader functional impairment. A high-complexity cluster comprised approximately 26.2% of referrals and was characterised by substantial psychiatric comorbidity, greater functional impairment and a higher likelihood of requiring differential diagnostic work involving autism, trauma-related disorders, mood disorders, substance misuse or personality-related difficulties. ADHD diagnosis rates were broadly similar across the three clusters. The clusters therefore represent gradations of presentation complexity rather than gradations of diagnostic likelihood.

The cluster proportions are used here as weighting factors in the base-case full ADHD assessment cost. The case-mix weighted mean is calculated as (0.302 × £850) + (0.436 × £1, 018) + (0.262 × £1, 186) = £1, 011.28. Under the service’s observed case mix, this weighted mean is the most empirically anchored single estimate of the average full ADHD assessment cost for adults referred to a specialist NHS adult ADHD service.

### 2.11 Characterising uncertainty

One-way sensitivity analyses were conducted on the principal cost drivers: full ADHD assessment cost, proportion screened out, CASQ duration, annual referral volume, and additional Band 7 clinical time for medium- and high-complexity cases.

Three resource-use scenarios were specified for the full ADHD assessment pathway. Scenario A is the standard or low-complexity pathway, costed at the £850 NHS England guide price. Scenario B is a medium-complexity pathway, costed as £850 plus two additional Band 7 client contact hours, giving a full ADHD assessment cost of £1, 018. Scenario C is a high-complexity pathway, costed as £850 plus four additional Band 7 client contact hours, giving a full ADHD assessment cost of £1, 186.

An expanded structural probabilistic sensitivity analysis was conducted using 10, 000 Monte Carlo simulations on the SWYPFT case-mix weighted-mean base case, with reproducibility seed 20260502, following established decision-modelling methods (35). Rather than sampling the weighted-mean full ADHD assessment cost as a single aggregate parameter, the expanded probabilistic sensitivity analysis separately propagated uncertainty in the SWYPFT complexity-cluster proportions, the incremental Band 7 clinical hours assigned to medium- and high-complexity full ADHD assessment cases, the CASQ assessment-and-triage cost, the £850 standard full ADHD assessment guide price and underlying Band 7 client contact hour cost, and the diagnostic accuracy parameters. Cost distributions were Gamma with mean £120.27 and SD £12.03 for the CASQ assessment-and-triage cost, Gamma with mean £850 and SD £127.50 for the standard or low-complexity full ADHD assessment guide price, and Gamma with mean £84 and SD £8.40 for the Band 7 client contact hour cost. Cluster proportions were sampled jointly from a Dirichlet distribution with parameters adjusted using a Jeffreys +0.5 offset: Dirichlet(237.5, 343.5, 206.5). Incremental Band 7 hours were sampled from triangular distributions with minimum, mode and maximum of 1, 2 and 3 hours for the medium-complexity scenario and 3, 4 and 5 hours for the high-complexity scenario. Diagnostic accuracy parameters were sampled using beta distributions: Beta(6.5, 42.5) for the observed within-sample ADHD case proportion, Beta(6.5, 0.5) for CASQ sensitivity, and Beta(19.5, 23.5) for CASQ specificity.

### 2.12 Approach to engagement with patients and others affected by the study

No formal patient or public involvement was undertaken in the preparation of this analysis. The research question is an NHS commissioning and service design question. Patient perspectives on CASQ screening, ADHD diagnosis and service access are relevant to implementation and are addressed qualitatively in the Discussion.

## 3 Results

### 3.1 Study parameters

Both columns of **Table 1** reflect the same validation sample; the full ADHD assessment column illustrates the counterfactual in which all 48 referrals proceed to full ADHD assessment. The unit costs, resource-use assumptions and sources applied throughout the analysis are summarised in **Table 2**.

**Table 1:** Baseline characteristics of the validation study population, n = 48.

Characteristic	CASQ-first pathway	Full ADHD assessment
Age, mean (SD), years	35.4 (11.2)	35.4 (11.2)
Female sex, n (%)	34 (70.8)	34 (70.8)
ADHD confirmed, n (%)	6 (12.5)	6 (12.5)
Prior mental health treatment, n (%)	37 (77.1)	37 (77.1)
Referral source: primary care, n (%)	48 (100.0)	48 (100.0)
CASQ triage outcome: Appropriate, n (%)	29 (60.4)	N/A
CASQ triage outcome: Not Appropriate, n (%)	19 (39.6)	N/A

**Table 2:** Unit costs, resource-use assumptions and sources.

Resource item	Unit	Quantity	Unit cost	Source
CASQ delivery: Band 7 clinician	Contact hour	1.0	£84	PSSRU 2024 (24)
CASQ outcome letter: Band 4 admin	Letter	1.0	£10	PSSRU 2024 (24)
Postage: patient and GP	Letter	2 letters	£1 each	Royal Mail 2025/26 tariff
Wider CASQ overhead	Per referral	1.0	£24.27	PSSRU 2024; **Appendix C**
CASQ assessment-and-triage cost	Per referral	1.0	£120.27	PSSRU 2024; **Appendix C**
Standard full ADHD assessment	Pathway	1.0	£850	NHS England 2026/27 guidance (23)
Medium-complexity full ADHD assessment	Pathway	1.0	£1, 018	NHS England plus PSSRU
High-complexity full ADHD assessment	Pathway	1.0	£1, 186	NHS England plus PSSRU
Weighted-mean full ADHD assessment	Pathway	1.0	£1, 011.28	NHS England, PSSRU and Shakeel et al. (33)

### 3.2 Primary cost-consequence results

The CASQ-first pathway achieved 100.0% sensitivity and 100.0% negative predictive value in the validation sample. No false negatives were observed, but the confidence interval remains wide because only six ADHD-positive cases were present. Under the SWYPFT case-mix weighted-mean base case, the CASQ-first pathway costs £731.08 per referral compared with £1, 011.28 per referral for full ADHD assessment, producing an incremental cost of −£280.20 per referral (**Table 3**). At the local projected annual volume of 2, 136 referrals, this translates to an estimated annual cost reduction of £598, 507.

**Table 3:** Cost-consequence analysis: diagnostic outcomes and costs per referral, SWYPFT case-mix weighted-mean base case.

Outcome	CASQ-first pathway	Full ADHD assessment	Incremental
Sensitivity, % (95% CI)	100.0 (61.0–100.0)	100.0	0.0 (−39.0 to 0.0)
Specificity, % (95% CI)	**45.2 (31.2–60.1)**	N/A	N/A
Negative predictive value, % (95% CI)	100.0 (83.2–100.0)	100.0	0.0 (−16.8 to 0.0)
False negatives per 1, 000 referrals	0 (0–49)	0	0 (0–49)
True negatives screened out per 1, 000 referrals	396 (270–537)	0	+396 (+270 to +537)
CASQ assessment-and-triage cost	£120.27	–	+£120.27
Full ADHD assessment, weighted mean	£610.81	£1, 011.28	−£400.47
Total cost per referral	£731.08	£1, 011.28	−£280.20
Annual cost at 2, 136 referrals	£1, 561, 587	£2, 160, 094	−£598, 507

The specificity confidence interval has been standardised to the value reported in the companion validation study (21).

Bold values indicate the CASQ specificity estimate and its 95% confidence interval, as reported in the companion validation study (21).

### 3.3 Probabilistic sensitivity analysis

In the expanded structural probabilistic sensitivity analysis on the SWYPFT case-mix weighted-mean base case, the CASQ-first pathway was cheaper in every one of 10, 000 simulations and met the specified 90% sensitivity threshold in 75.0% of simulations (**Table 4**). In 25.0% of simulations sensitivity fell below the specified threshold. The probabilistic mean saving was £285.18 per referral and £609, 143 annually at 2, 136 referrals. Because the CASQ-first pathway was cost-saving in 100.0% of simulations under the specified assumptions, the residual 25.0% risk is a diagnostic-safety risk, not an economic risk. This separation should be read as a reminder that, although the cost saving is robust to the modelled cost uncertainty, it remains conditional on a sensitivity estimate derived from only six confirmed ADHD cases; the economic conclusion is therefore no more secure than the underlying diagnostic-safety evidence.

**Table 4:** Expanded structural probabilistic sensitivity analysis results, SWYPFT case-mix weighted-mean base case.

Parameter	Mean estimate (95% CrI)
CASQ sensitivity	92.9% (67.1–100.0%)
CASQ specificity	45.3% (31.0–60.1%)
Within-sample ADHD case proportion	13.2% (5.4–23.8%)
Low-complexity cluster proportion	30.2% (27.0–33.4%)
Medium-complexity cluster proportion	43.6% (40.2–47.1%)
High-complexity cluster proportion	26.2% (23.2–29.3%)
CASQ assessment-and-triage cost per referral	£120.33 (£98.29–£144.91)
Weighted-mean full ADHD assessment cost	£1, 007.04 (£776.71–£1, 272.75)
False negatives per 1, 000 referrals	9.4 (0.0–47.3)
True negatives screened out per 1, 000 referrals	393.4 (264.4–531.3)
CASQ-first pathway cost per referral	£721.86 (£532.03–£942.44)
Full ADHD assessment cost per referral	£1, 007.04 (£776.71–£1, 272.75)
Incremental cost per referral	−£285.18 (−£472.54 to −£130.39)
Annual saving at 2, 136 referrals	£609, 143 (£278, 514–£1, 009, 355)
Probability CASQ-first pathway is cost-saving	100.0%
Probability CASQ sensitivity ≥ 90%	75.0%
Probability cost-saving and sensitivity ≥ 90%	75.0%

### 3.4 Deterministic sensitivity and scenario analyses

Across the component scenarios for the standard, medium- and high-complexity profiles, and across all the sensitivity analyses listed in **Table 5**, the CASQ-first pathway remains cost-saving against full ADHD assessment, with the single exception of the deliberately conservative lower-bound symmetrical staff-cost stress test.

**Table 5:** Deterministic sensitivity and scenario analyses.

Analysis	Full cost	CASQ cost	Saving	Annual saving
Base case: weighted mean	£1, 011.28	£731.08	£280.20	£598, 507
Standard or low-complexity	£850	£633.67	£216.33	£462, 081
Medium-complexity	£1, 018	£735.14	£282.86	£604, 189
High-complexity	£1, 186	£836.61	£349.39	£746, 297
Assessment cost −20%	£809.02	£608.92	£200.10	£427, 414
Assessment cost +20%	£1, 213.54	£853.25	£360.29	£769, 579
Screened out lower CI	£1, 011.28	£858.50	£152.78	£326, 338
Screened out upper CI	£1, 011.28	£588.49	£422.79	£903, 080
CASQ duration 45 min	£1, 011.28	£710.08	£301.20	£643, 363
CASQ duration 75 min	£1, 011.28	£752.08	£259.20	£553, 651
Annual volume 1, 000 referrals	£1, 011.28	£731.08	£280.20	£280, 200
Annual volume 2, 500 referrals	£1, 011.28	£731.08	£280.20	£700, 500
Staff-cost stress test	£168	£221.74	−£53.74	−£114, 789

### 3.5 Conditional cost-minimisation scenario

This scenario is presented secondary to the primary cost-consequence analysis and should be interpreted only in commissioning contexts where diagnostic non-inferiority is accepted *a priori*. Under the assumption that CASQ sensitivity is equivalent to full ADHD assessment, the deterministic SWYPFT case-mix weighted-mean base case estimates that the CASQ-first pathway generates a saving of £280.20 per referral. For the 39.6% of referrals classified as Not Appropriate, the £1, 011.28 weighted-mean full ADHD assessment is avoided and replaced by the £120.27 CASQ assessment-and-triage cost, saving £891.01 per screened-out referral. For the 60.4% of referrals classified as Appropriate, the CASQ adds £120.27 to the cost of full ADHD assessment.

### 3.6 Commissioner-facing budget-impact interpretation

Although the primary economic unit in this analysis is cost per referral, commissioners are more likely to interpret the findings in annual budget-impact terms. Under the SWYPFT case-mix weighted-mean base case, the CASQ-first pathway saves £280.20 per referral. This means that every 1, 000 referrals managed through the CASQ-first pathway would be expected to reduce assessment-pathway expenditure by approximately £280, 200, assuming that the observed screening-out rate, cost structure and case-mix assumptions apply.

At the projected local annual volume of 2, 136 referrals, this translates into an estimated annual saving of £598, 507. The corresponding annual saving is £462, 081 under the standard or low-complexity scenario, £604, 189 under the medium-complexity scenario, and £746, 297 under the high-complexity scenario. These figures are more directly interpretable for commissioners because they express the effect of CASQ implementation as a service-level budget impact rather than only as a per-referral cost difference.

## 4 Discussion

### 4.1 Principal findings and their meaning

This cost-consequence analysis suggests that a CASQ-first pathway may reduce the cost of managing adults referred for specialist ADHD assessment by redirecting a substantial proportion of referrals away from immediate full ADHD assessment, when these are not clinically appropriate for such assessment. In the SWYPFT case-mix weighted-mean base case, the pathway generated an estimated saving of £280.20 per referral, equivalent to £598, 507 at a projected local annual volume of 2, 136 referrals. The headline base case uses a CASQ assessment-and-triage cost of £120.27 per referral and a weighted-mean full ADHD assessment cost of £1, 011.28 calculated from the same service’s observed case-mix proportions.

The more important finding is that cost saving and diagnostic safety are supported by different levels of certainty. The cost result is relatively stable because it is driven by a clear resource mechanism. Diagnostic safety, however, is less certain, as the validation study included only 48 participants and six confirmed ADHD cases. Although CASQ identified all ADHD-positive cases in that sample, the confidence interval around sensitivity remains wide. The observed finding is encouraging, but it does not yet provide sufficiently precise evidence that a CASQ-first pathway has diagnostic safety comparable with a pathway in which all referred individuals proceed directly to full ADHD assessment. Accordingly, the economic conclusions reported here remain conditional upon confirmation of diagnostic safety in larger, independent samples and should not be read as established savings.

The expanded structural probabilistic sensitivity analysis makes this distinction clearer: the CASQ-first pathway was cost-saving in 100.0% of simulations but met the specified 90% sensitivity threshold in only 75.0% of simulations. In 25.0% of simulations sensitivity fell below the specified threshold. The residual 25.0% risk is therefore a diagnostic-safety risk, not an economic risk.

### 4.2 Economic interpretation

ADHD assessment capacity is scarce, costly and increasingly consumed by referrals with varying diagnostic probability. If every person referred automatically receives a full ADHD assessment then such a pathway treats every referral as requiring the same specialist diagnostic resource. A CASQ-first pathway changes that allocation rule by introducing an intermediate clinical decision point. The pathway creates economic value only if triage identifies a meaningful group of referrals that do not require immediate full ADHD assessment.

In the SWYPFT case-mix weighted-mean base case, 39.6% of referrals were classified as Not Appropriate for full ADHD assessment. In the model, these referrals incur the £120.27 CASQ assessment-and-triage cost rather than the £1, 011.28 weighted-mean full ADHD assessment pathway, a per-referral avoided cost of £891.01 for those screened out. The saving generated by this group is large enough to offset the additional CASQ overhead incurred for the 60.4% of referrals who still proceed to full ADHD assessment.

The capacity argument may matter more to commissioners than the cost argument. At the local projected activity volume of 2, 136 referrals, a 39.6% screening-out rate translates into approximately 846 referrals diverted from full ADHD assessment annually. Capacity released is the operationally meaningful quantity because it is what allows services to reduce waiting times for the patients who do require full ADHD assessment. This interpretation is consistent with the wider economic literature on adult ADHD, which documents substantial treatment-related economic effects (36), impaired health-related quality of life and work productivity (37), long-term fiscal consequences of the disorder (38) and health-state utilities suitable for future model-based evaluations (39).

### 4.3 Why cost-consequence analysis is the correct framework

An economic analysis built on diagnostic-accuracy inputs from an underpowered validation study cannot, in itself, justify implementation. The present analysis is therefore not a definitive economic case for implementation of CASQ, but a structured exploration of what the economics would look like if the diagnostic safety margin were confirmed in an adequately powered multi-site study. The cost-consequence framework is justified because the two main outcomes, cost reduction and diagnostic safety, cannot yet be collapsed into a single conclusion. A cost-minimisation analysis would require sufficient evidence that the CASQ-first pathway and full ADHD assessment produce equivalent diagnostic outcomes at pathway level. That condition has not yet been met, because the number of ADHD-positive cases in the validation study was too small to establish non-inferiority with adequate precision.

### 4.4 Complexity, case mix and the economics of triage

Adult ADHD referrals are clinically heterogeneous, and some cases can be assessed within the standard pathway represented by the £850 NHS England guide price. Others require additional clinical time because of diagnostic uncertainty, multiple psychiatric comorbidities, complex medication review, risk or safeguarding concern, or differential diagnosis involving autism, bipolar disorder, psychosis, trauma-related disorders, substance misuse or personality-related difficulties. The complexity-based scenarios model this by adding incremental Band 7 client contact hours to the £850 commissioning anchor: two additional hours for the medium-complexity scenario and four additional hours for the high-complexity scenario.

The complexity-stratified scenarios are supported by the previous explainable clustering analysis of 786 consecutive adult ADHD referrals to the same service (33). The scenarios show that as full ADHD assessment cost rises, the saving generated by safe triage rises with it. Component B, costed at £1, 018, yields a per-referral saving of £282.86 and an annual saving of £604, 189. Component C, costed at £1, 186, yields £349.39 per referral and £746, 297 annually. The structural argument is independent of the precise additional clinical-time assumption: a flat tariff under-rewards services that absorb a higher-complexity case mix, and any mechanism that protects assessment capacity from low-yield referrals will tend to generate larger savings in those services.

### 4.5 Diagnostic safety, false negatives and the threshold for adoption

The main clinical risk is the false negative, meaning a person with ADHD who is classified as Not Appropriate and therefore does not proceed to full ADHD assessment. No false negatives were observed in the validation study, which is reassuring. However, the validation sample contained only six confirmed ADHD cases. The absence of observed false negatives in such a small sample cannot establish that the false-negative rate is negligible in routine practice.

The expanded structural probabilistic sensitivity analysis provides a more realistic account of that uncertainty. The mean projected false-negative rate was 9.4 per 1, 000 referrals, but the credible interval extended to 47.3 per 1, 000. The lower end of that range is reassuring; the upper end would be clinically important. In a high-volume service, even a low false-negative rate can translate into a meaningful number of missed or delayed diagnoses. At scale, small errors in triage sensitivity become large absolute numbers.

The adoption threshold should therefore be set by diagnostic safety, not by cost saving. The CASQ-first pathway should be considered ready for broader implementation only when a larger study shows that the pathway-level false-negative risk is acceptably low. That study should use consecutive referrals, blinded full ADHD assessment for all participants, a specified non-inferiority margin and sufficient numbers of confirmed ADHD cases.

### 4.6 Equity and implementation safeguards

Screening out a referral does not mean that need is absent. It means that ADHD was not judged to be the appropriate explanatory framework or assessment pathway at that point. Individuals classified as Not Appropriate may still have clinically significant anxiety, depression, trauma-related difficulties, autism, sleep disturbance, substance use, personality-related difficulties, cognitive impairment or social adversity. A triage pathway is defensible only if those needs are recognised and redirected appropriately. In the validation service, referrals classified as Not Appropriate were discharged from the ADHD pathway and signposted to appropriate alternative support rather than retained without action (21).

Implementation should therefore include safeguards. Patients and referrers should receive clear written explanations of the triage decision. There should be explicit signposting to appropriate alternative services. Borderline and complex cases should have access to senior clinical review. New information should trigger reconsideration. Services should monitor re-referral rates, subsequent ADHD diagnoses after an initial Not Appropriate outcome, complaints, demographic patterns in screening decisions and patient-reported experience.

### 4.7 Strengths and limitations

The main strength of this analysis is that it is anchored in a validation study in which all participants received full ADHD assessment regardless of CASQ triage outcome. This reduces verification bias, a common weakness in triage and screening studies. The analysis also uses published NHS and PSSRU cost sources, separates costs from diagnostic outcomes, and avoids a premature cost-minimisation claim. The expanded structural probabilistic sensitivity analysis propagates joint uncertainty in the SWYPFT complexity-cluster proportions, the incremental Band 7 clinical hours for medium- and high-complexity full ADHD assessments, the CASQ assessment-and-triage cost, the standard full ADHD assessment guide price and the Band 7 hourly cost, and the diagnostic accuracy parameters.

The principal limitation is the small, underpowered validation sample. The diagnostic accuracy inputs are derived from a single-site study with 48 participants and only six confirmed ADHD cases, which is well below the approximately 179 confirmed ADHD cases, or 1, 432 consecutive referrals at the observed rate of positive diagnosis, required to test the −10 percentage-point non-inferiority margin under standard assumptions. The economic conclusions are therefore explicitly conditional on a future adequately powered study confirming the diagnostic-safety margin within which the present cost estimates are valid. They are presented as commissioning planning figures, not as established cost savings.

### 4.8 Generalisability of the diagnostic and economic inputs

The diagnostic and economic inputs are drawn from a single NHS service, and several features can affect their transferability. First, the 12.5% within-sample diagnostic rate is an artefact of the universal-assessment validation design, in which every referral received full ADHD assessment, including those who would ordinarily have been screened out; it is therefore lower than the diagnostic rate that a service would observe in routine operation and should not be interpreted as a representative service diagnostic rate. Second, services differ in referral quality, case mix, comorbidity burden and diagnostic thresholds, and these differences would influence both CASQ screening performance and the projected savings. For example, a service with a higher diagnostic rate or a lower screened-out proportion would realise smaller per-referral savings, while the converse would apply where a larger share of referrals is appropriately screened out. Finally, the present analysis adopts an NHS secondary-care perspective, whereas a substantial proportion of adult ADHD diagnostic activity in England is currently delivered by independent providers under Right to Choose arrangements so the transferability of these NHS-perspective estimates to that mixed provision landscape requires separate consideration.

### 4.9 Implications for commissioners and future research

For commissioners, the CASQ-first pathway offers a potentially important response to rising ADHD assessment demand. It provides a structured method for separating referrals that appear to require full specialist assessment from those more appropriately directed elsewhere. If diagnostic safety is confirmed, this could improve the allocation of scarce specialist capacity, reduce unnecessary full ADHD assessments, shorten waiting lists and improve access for those whose presentation warrants full specialist assessment.

However, the current evidence supports cautious, governed implementation rather than unconditional adoption. CASQ should not be implemented simply as a demand-reduction tool. It should be implemented, if at all, as a clinically governed pathway-allocation process with explicit monitoring of false negatives, re-referrals, patient experience and equity. Commissioners should also ensure that alternative pathways are available for people screened out of ADHD assessment. Without such alternatives, triage risks becoming exclusion rather than appropriate redirection.

The next research step should be an independent, multi-site, prospective non-inferiority study. Independent external validation, conducted by research groups not involved in the development of CASQ, is particularly important before wider implementation, given that the diagnostic-accuracy inputs underpinning this analysis derive from the developing group’s own validation work. It should enrol consecutive referrals, apply CASQ before full ADHD assessment, ensure all participants receive blinded full ADHD assessment, and include prospective micro-costing of both pathways using comparable resource categories. It should be powered around confirmed ADHD cases, approximately 179 confirmed cases under the assumptions used here, corresponding to approximately 1, 432 consecutive referrals at the observed rate of positive diagnosis. It should include longitudinal follow-up of those classified as Not Appropriate.

### 4.10 Illustrative national-scale implications

The local budget-impact findings indicate that the potential aggregate implications of a CASQ-first pathway could be substantial if the pathway were replicated safely across services. At the projected local annual volume of 2, 136 referrals, the SWYPFT case-mix weighted-mean base-case saving was £598, 507, based on a saving of £280.20 per referral. In operational terms, the same screening-out rate of 39.6% would mean that approximately 846 referrals per year would not proceed to full ADHD assessment, releasing specialist assessment capacity for those whose presentation more clearly warrants full diagnostic assessment.

The following national figures are presented as illustrative policy scenarios to indicate the possible order of magnitude of savings and capacity release and are not established national savings and should not be cited as such. NHS England Management Information recorded approximately 16, 100 new monthly referrals for suspected ADHD in December 2024, equivalent to approximately 193, 000 referrals across the twelve months ending December 2024, and approximately 21, 150 new referrals per month on average across calendar year 2025, equivalent to approximately 254, 000 referrals across that calendar year (5). Applying the SWYPFT case-mix weighted-mean saving of £280.20 per referral to these referral-volume anchors gives an illustrative national saving of approximately £54.1 million at 193, 000 referrals and approximately £71.2 million at 254, 000 referrals.

These national figures are illustrative and should not be interpreted as definitive national budget-impact estimates. They show the order of magnitude of potential savings and capacity release if the observed CASQ screening-out rate, cost structure and diagnostic safety profile were reproduced across wider NHS services. Realised savings would depend on local referral quality, case mix, diagnostic thresholds, staffing model, clinical governance, alternative pathway availability and whether released capacity is converted into cash-releasing savings, reduced waits or additional activity.

## 5 Conclusion

This cost-consequence analysis estimates that, under the modelled assumptions, the CASQ-first pathway would reduce the cost of managing adults referred for specialist ADHD assessment compared with a full ADHD assessment, from an NHS commissioning perspective. The findings are preliminary and conditional on future adequately powered multi-site validation confirming diagnostic non-inferiority. In the validation study, CASQ achieved 100% sensitivity, but the study included only 48 participants and only six confirmed ADHD cases. The 95% confidence interval for sensitivity was wide, and the validation study was not adequately powered to test a formal non-inferiority margin of −10 percentage points.

Under the SWYPFT case-mix weighted-mean base case, with a CASQ assessment-and-triage cost of £120.27 per referral and a weighted-mean full ADHD assessment cost of £1, 011.28 per referral, the CASQ-first pathway was estimated to save £280.20 per referral and £598, 507 annually at 2, 136 referrals. In the expanded structural probabilistic sensitivity analysis, the CASQ-first pathway was cost-saving in 100.0% of simulations and met the specified 90% sensitivity threshold in 75.0% of simulations. Because the pathway was cost-saving in 100.0% of simulations under the specified assumptions, the residual uncertainty is wholly diagnostic. The economic result is stronger than the current evidence for diagnostic safety. The per-referral and national figures reported here are planning and policy scenarios conditional on future confirmation of diagnostic safety, not established savings.

The conditional cost-minimisation scenario, presented secondarily to the primary cost-consequence analysis, suggests that if diagnostic non-inferiority is replicated in adequately powered multi-site studies, the CASQ-first pathway would be economically attractive. However, a cost-saving pathway is not necessarily a diagnostically safe pathway. CASQ is a pathway-allocation intervention and not a substitute for full ADHD assessment. Commissioners considering CASQ should ensure that implementation is accompanied by monitoring of clinical outcomes and equity indicators, adequate resources for signposting and alternative care pathways, formal patient experience measurement and contingency planning for false negatives.

## Data Availability

The original contributions presented in the study are included in the article/Supplementary Material. Further inquiries can be directed to the corresponding author.
