# Supplementary material for: Cost-consequence analysis of a criterion-based screening and triage pathway compared with full ADHD assessment for adults referred to NHS secondary care
Source: Front Psychiatry. 2026 Aug 3;17:1884417. doi: 10.3389/fpsyt.2026.1884417 (PMC13480067; doi:10.3389/fpsyt.2026.1884417)
Supplement: Supplementary file 1 [file SupplementaryFile1.docx]

# Appendix A. Non-inferiority sample size calculation

The validation study observed six true positives among six confirmed ADHD cases, corresponding to observed sensitivity of 100%. However, diagnostic non-inferiority depends on the number of confirmed ADHD cases rather than the total number of referrals. The relevant statistical problem is therefore an approximate one-sample binomial non-inferiority calculation for sensitivity. The null and alternative hypotheses were specified as H0: sensitivity ≤ 0.90 and H1: sensitivity > 0.90.

The non-inferiority threshold was 90% sensitivity, corresponding to a maximum acceptable false-negative rate of 10%. The one-sided alpha level was 0.05. The target power was 80%. The anticipated true sensitivity for planning purposes was 95%. The expected rate of positive diagnosis in the referred population was 12.5%, based on the observed rate in the validation study. Under these assumptions, the required number of confirmed ADHD cases is approximately 179, and the total referral sample size is N = 179 / 0.125 ≈ 1,432.

**Table S1. Non-inferiority sample size assumptions.**

| **Assumption** | **Value** |
| --- | --- |
| Test | Approximate one-sample binomial non-inferiority calculation |
| Null hypothesis | Sensitivity ≤ 90% |
| Alternative hypothesis | Sensitivity > 90% |
| Alpha | 0.05, one-sided |
| Target power | 80% |
| Anticipated true sensitivity | 95% |
| Rate of positive diagnosis | 12.5% |
| Confirmed ADHD cases required | Approximately 179 |
| Total referrals required | Approximately 1,432 |

# Appendix B. CHEERS 2022 reporting compliance

**Table S2. CHEERS 2022 reporting compliance.**

| **#** | **CHEERS 2022 item** | **Where addressed** |
| --- | --- | --- |
| 1 | Title | Title page |
| 2 | Structured abstract | Abstract |
| 3 | Background and objectives | Section 1 |
| 4 | Health economic analysis plan | Section 2.1 |
| 5 | Study population | Section 2.2 |
| 6 | Setting and location | Section 2.3 |
| 7 | Comparators | Section 2.4 |
| 8 | Perspective | Section 2.5 |
| 9 | Time horizon | Section 2.5 |
| 10 | Discount rate | Section 2.5 |
| 11 | Selection of outcomes | Section 2.6 |
| 12 | Measurement of outcomes | Sections 2.6–2.11 |
| 13 | Valuation of outcomes | Not applicable; diagnostic outcomes were not valued monetarily |
| 14 | Measurement and valuation of resources and costs | Sections 2.7–2.8 |
| 15 | Currency, price date and conversion | Section 2.8 |
| 16 | Rationale and description of model | Section 2.9 |
| 17 | Analytics and assumptions | Sections 2.9–2.11 |
| 18 | Characterising heterogeneity | Section 2.10 |
| 19 | Characterising distributional effects | Discussion |
| 20 | Characterising uncertainty | Section 2.11 |
| 21 | Patient engagement | Section 2.12 |
| 22 | Study parameters | Section 3.1 |
| 23 | Summary of main results | Sections 3.2–3.5 |
| 24 | Effect of uncertainty | Sections 3.3–3.4 |
| 25 | Effect of engagement | Discussion |
| 26 | Discussion | Section 4 |
| 27 | Funding | Funding statement |
| 28 | Conflicts of interest | Conflict of interest statement |

# Appendix C. Bottom-up itemisation of the CASQ assessment-and-triage cost

**Table S3. Per-referral cost components, CASQ assessment-and-triage cost.**

| **Cost component** | **Assumption** | **Cost** |
| --- | --- | --- |
| CASQ delivery | 1 Band 7 client contact hour at £84/hour | £84.00 |
| Outcome letter, admin | Approximately 16 min Band 4 admin at £38/hour | £10.00 |
| Outcome letter, postage | 2 letters × £1 each | £2.00 |
| Referral administration | 10 min Band 4 admin at £38/hour | £6.33 |
| Appointment booking | 10 min Band 4 admin at £38/hour | £6.33 |
| Clinical supervision | 1 consultant psychiatrist hour/week at £157/hour, allocated across 2,136 referrals | £3.82 |
| Clinical governance | 12 senior staff hours/year at £86/hour | £0.48 |
| Audit and quality assurance | 10% of referrals × 30 min Band 7 | £3.30 |
| Information governance | 4 senior staff hours/year at £86/hour | £0.16 |
| Non-attendance management | 10% of referrals × 10 min Band 4 admin | £0.63 |
| Managerial overhead | 24 senior staff hours/year at £86/hour | £0.97 |
| Initial training | £1,973 one-off cost annualised | £0.92 |
| Refresher training | £1,156 annual cost | £0.54 |
| Service set-up | £1,645 one-off cost annualised | £0.77 |
| Estates | Already included in PSSRU staff overheads | £0.00 |
| Digital infrastructure | Local business cases should add where relevant | £0.00 |
| **CASQ assessment-and-triage cost** |  | **£120.27** |

Bold values indicate the total CASQ assessment-and-triage cost per referral. The total was calculated from unrounded component values; displayed components may not sum exactly because they are rounded to two decimal places.
